# Supplementary material for: Using the Rasch Model to Understand Consumers’ Behaviour in Buying Kiwifruits
Source: Foods. 2025 Jul 30;14(15):2683. doi: 10.3390/foods14152683 (PMC12346770; doi:10.3390/foods14152683)
Supplement: Supplementary file 1 [file foods-14-02683-s001.zip › foods-3720216-supplementary.pdf]

Supplement Figure S1. Kiwi Attitude: Wright Map.

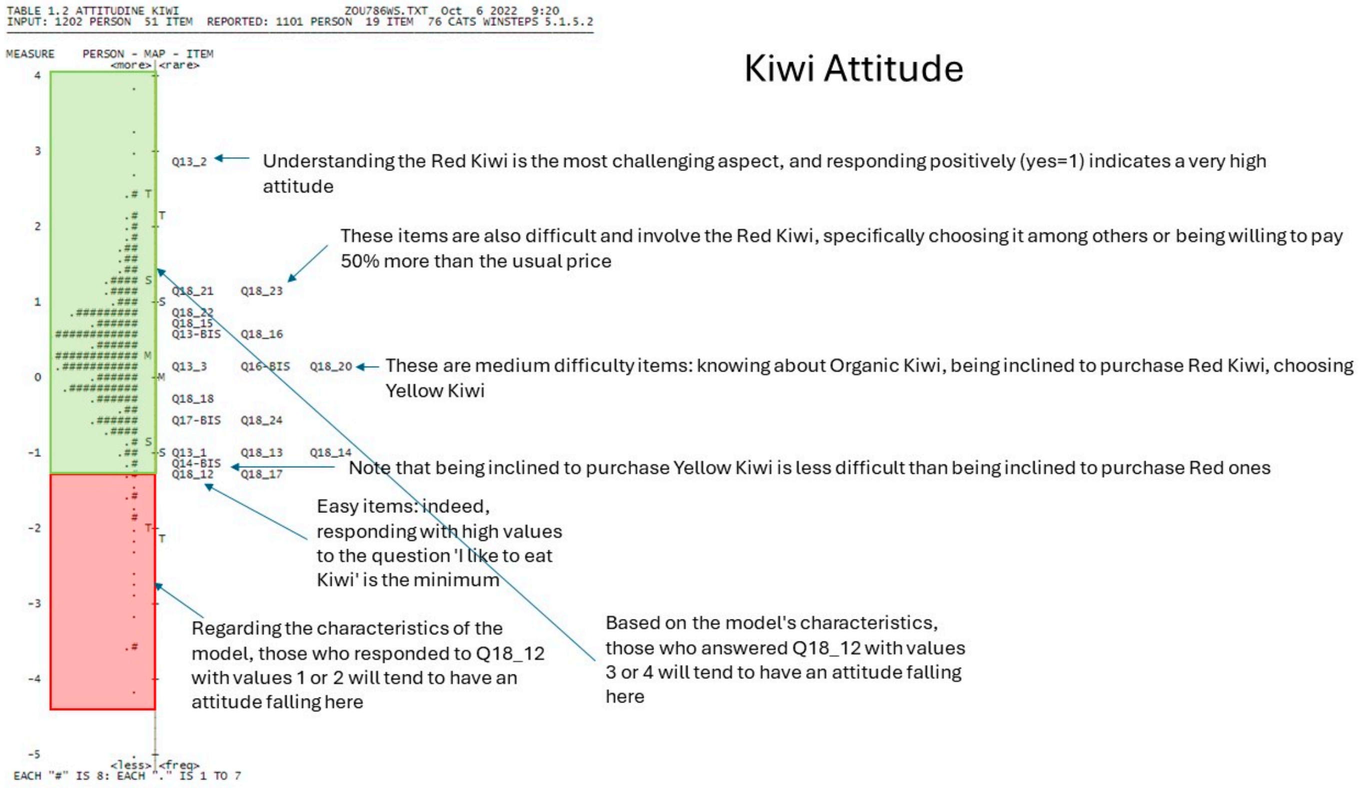

Supplement Figure S2. Categories of items positioned at the highest probability of attitude value.

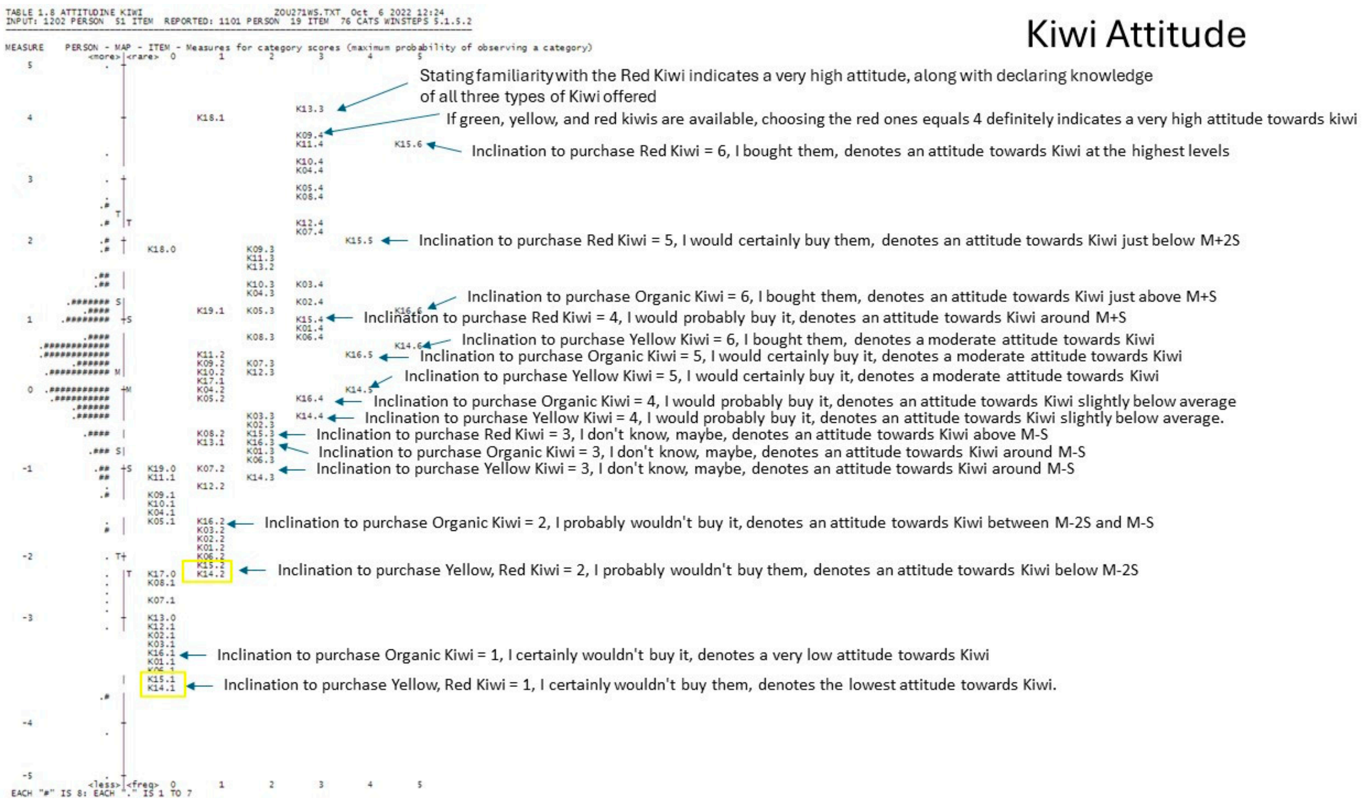

Supplement Table S1. Items related to consumer attitude: level of agreement/disagreement (%).

	Agreement (totally and partially)					Disagreement (totally and partially)				
	IT	ES	FR	DE	TOT	IT	ES	FR	DE	TOT
Fresh fruit is a very healthy product	23.7	23.7	23.9	23.7	**95.0**	1.2	1.3	1.2	1.2	**5.0**
I like to eat fresh fruit.	23.8	24.0	23.3	23.2	**94.3**	1.2	1.0	1.7	1.7	**5.7**
I eat fresh fruit because it's good for my health.	23.8	23.8	23.6	23.0	**94.3**	1.2	1.2	1.4	1.9	**5.7**
Fresh fruit provides a variety of nutrients.	23.5	23.7	22.9	23.4	**93.5**	1.4	1.3	2.2	1.6	**6.5**
I enjoy eating fresh fruit as it is. without any special transformations.	23.2	23.4	22.5	22.6	**91.8**	1.7	1.7	2.5	2.3	**8.2**
I enjoy eating kiwi as it is.	21.8	22.5	22.5	21.6	**88.4**	3.2	2.6	2.6	3.3	**11.6**
For me. it's essential to eat fresh fruit every day.	22.5	21.8	22.5	20.5	**87.4**	2.4	3.2	2.5	4.4	**12.6**
I enjoy eating kiwis.	21.4	21.4	22.5	21.3	**86.6**	3.6	3.7	2.5	3.7	**13.4**

I eat kiwi for its remarkable nutritional properties.	21.1	21.9	22.0	20.0	**85.0**	3.8	3.2	3.0	5.0	**15.0**
I eat kiwi for its high content of vitamin C.	21.3	20.9	21.1	20.0	**83.4**	3.7	4.2	3.9	4.9	**16.6**
I enjoy eating fresh fruit as an ingredient in cakes. fruit salads. smoothies. etc.	20.9	19.5	21.0	19.0	**80.3**	4.1	5.6	4.1	6.0	**19.7**
I enjoy experimenting with new foods and flavours. even if I'm not familiar with them.	19.1	19.1	19.2	15.6	**72.9**	5.9	6.0	5.8	9.4	**27.1**
If green. yellow. and red kiwis are available. I choose the green ones.	18.9	18.8	17.1	17.6	**72.5**	6.1	6.2	7.9	7.3	**27.5**
I enjoy eating kiwi as an ingredient in cakes. fruit salads. smoothies. etc.	18.9	17.4	17.0	15.8	**69.1**	6.1	7.7	8.1	9.2	**30.9**
I am willing to follow a fruit-based diet for 1 day.	16.1	14.8	15.7	14.6	**61.3**	8.8	10.2	9.3	10.3	**38.7**
If green. yellow. and red kiwis are available. I choose the yellow ones.	12.8	15.1	14.5	12.6	**55.0**	12.1	9.9	10.6	12.4	**45.0**
I usually buy fresh fruit only if I find it at a low price.	12.4	10.6	12.6	14.0	**49.6**	12.6	14.4	12.5	11.0	**50.4**
I am willing to follow a fruit-based diet for 1 week.	13.2	11.7	11.9	9.6	**46.4**	11.7	13.3	13.1	15.4	**53.6**
I eat kiwi for its laxative effect.	13.0	16.4	9.6	6.5	**45.4**	12.0	8.7	15.5	18.5	**54.6**
If I don't know a food. I don't eat it.	10.8	11.7	8.5	10.1	**41.1**	14.1	13.3	16.6	14.9	**58.9**
I eat kiwi (after dinner) because it improves the quality of my sleep.	10.6	10.7	11.7	6.0	**39.1**	14.3	14.3	13.3	19.0	**60.9**
I am willing to pay 25% more than the usual price to try a red kiwi.	10.3	9.2	9.2	8.9	**37.6**	14.6	15.9	15.8	16.1	**62.4**
If green. yellow. and red kiwis are available. I choose the red ones.	8.7	7.7	6.3	6.4	**29.1**	16.2	17.4	18.7	18.6	**70.9**
I am willing to pay 50% more than the usual price to try a red kiwi.	7.9	8.0	7.3	5.2	**28.4**	17.1	17.1	17.7	19.8	**71.6**
I usually don't buy fresh fruit because it's too expensive.	6.2	6.0	6.2	5.1	**23.4**	18.8	19.1	18.9	19.9	**76.6**

Supplement Table S2. Items for measuring ATTITUDE TOWARD KIWI.

NAME	ENTRY	CODE	DESCRIPTION	VALUES			
K01	31	Q18_12	I like eating kiwi.	1 = completely disagree	2	3	4 = completely agree
K02	32	Q18_13	I eat kiwi for its remarkable nutritional properties.	1 = completely disagree	2	3	4 = completely agree
K03	33	Q18_14	I eat kiwi for its high vitamin C content.	1 = completely disagree	2	3	4 = completely agree
K04	34	Q18_15	I eat kiwi (after dinner) because it improves the quality of my sleep.	1 = completely disagree	2	3	4 = completely agree
K05	35	Q18_16	I eat kiwi for its laxative effect.	1 = completely disagree	2	3	4 = completely agree
K06	36	Q18_17	I enjoy eating kiwi as it is.	1 = completely disagree	2	3	4 = completely agree
K07	37	Q18_18	I enjoy eating kiwi as an ingredient in cakes, fruit salads, smoothies, etc.	1 = completely disagree	2	3	4 = completely agree
K08	39	Q18_20	If green, yellow, and red kiwis are available, I choose the yellow ones.	1 = completely disagree	2	3	4 = completely agree
K09	40	Q18_21	If green, yellow, and red kiwis are available, I choose the red ones.	1 = completely disagree	2	3	4 = completely agree
K10	41	Q18_22	I am willing to pay 25% more than the usual price to try a red kiwi.	1 = completely disagree	2	3	4 = completely agree
K11	42	Q18_23	I am willing to pay 50% more than the usual price to try a red kiwi.	1 = completely disagree	2	3	4 = completely agree
K12	43	Q18_24	I enjoy experimenting with new foods and flavors, even if I'm not familiar with them.	1 = completely disagree	2	3	4 = completely agree
K13	45	Q13-BIS	How many kiwi varieties do you know between YELLOW, RED, and ORGANIC?	0, 1, 2, 3			
K14	46	Q14-BIS	Propensity to purchase Yellow Kiwi	1 = Certainly would not purchase, 2 = Probably would not purchase, 3 = Unsure, 4 = Probably would purchase, 5 = Certainly would purchase, 6 = Already purchased			
K15	47	Q16-BIS	Propensity to purchase Red Kiwi				
K16	48	Q17-BIS	Propensity to purchase Organic Kiwi				
K17	49	Q13_1	Are you familiar with Yellow Kiwi?	0=NO, 1=YES			
K18	50	Q13_2	Are you familiar with Red Kiwi?				
K19	51	Q13_3	Are you familiar with Organic Kiwi?				

SOCIOECONOMIC CHARACTERISTICS OF THE SAMPLE

The survey involved a sample of 1202 respondents. equally distributed from Italy. Spain. France. and Germany (Italy and Germany: 300 units. France and Spain: 301). The responses provided by the interviewees were analysed using descriptive statistical techniques. The frequency values reported in the tables are percentages unless otherwise indicated. The following tables show the distribution of the Italian sample by region of origin and the breakdown of the entire sample by:

- Gender
- Age groups
- Education level
- Monthly income class
- Occupation.

Supplement Table S3. Sample distribution by GENDER.

	Italy	Spain	France	Germany	Total
Female	52.0	51.2	52.2	52.7	52.0
Male	48.0	48.5	47.5	47.3	47.8
Others	0.0	0.3	0.3	0.0	0.2
**Total**	**100.0**	**100.0**	**100.0**	**100.0**	**100.0**

Supplement Table S4. Sample distribution by AGE GROUPS.

	Italy	Spain	France	Germany	Total
18-24	8.7	8.6	11.3	8.3	9.2
25-34	15.3	15.6	16.3	14.3	15.4
35-44	19.3	20.3	18.3	17.0	18.7
45-54	18.0	18.6	17.3	18.7	18.1
55+	38.7	36.9	36.9	41.7	38.5
**Total**	**100.0**	**100.0**	**100.0**	**100.0**	**100.0**

Supplement Table S5. Sample distribution by EDUCATIONAL LEVEL.

	Italy	Spain	France	Germany	Total
Elementary school. lower middle school.	33.0	15.9	11.6	10.0	17.6
High school. vocational institute.	47.7	38.9	52.8	54.3	48.4
University or higher degree (e.g. doctorate).	19.3	45.2	35.5	35.7	33.9
**Total**	**100.0**	**100.0**	**100.0**	**100.0**	**100.0**

Supplement Table S6. Sample distribution by MONTHLY HOUSEHOLD INCOME CLASS.

	Italy	Spain	France	Germany	Total
Until 1000 EUR	15.3	7.0	26.9	23.0	18.1
Until 1500 EUR	26.0	18.3	19.9	15.7	20.0
Until 2000 EUR	22.0	23.9	15.6	20.0	20.4
Until 2500 EUR	12.0	20.6	13.0	13.0	14.6
Until 3000 EUR	13.0	7.6	8.3	8.7	9.4
More than 3000 EUR	11.7	22.6	16.3	19.7	17.6
**Total**	**100.0**	**100.0**	**100.0**	**100.0**	**100.0**

Supplement Table S7. Sample distribution by OCCUPATION.

	Italy	Spain	France	Germany	Total
Self-employed/merchant.	0.7	1.7	2.3	6.3	2.7
Entrepreneur.	0.7	3.0	0.7	2.3	1.7
Executive.	2.7	2.3	2.0	3.7	2.7
Official/Manager.	2.3	4.0	11.0	2.0	4.8
Employee/Teacher.	18.3	20.9	23.3	25.7	22.0
Worker/Salesperson/Dependent Farmer.	18.0	3.0	6.3	5.7	8.2
Freelancer.	4.3	18.3	1.7	5.3	7.4
Another self-employed worker.	2.3	1.3	2.0	2.0	1.9
Artisan.	0.7	1.3	1.7	5.3	2.2
Homemaker	11.0	4.3	7.3	7.3	7.5
Student	6.3	5.6	6.3	3.0	5.3
Retiree.	17.7	14.3	23.3	24.0	19.8
Unemployed/Seeking employment.	13.0	15.3	7.0	3.7	9.7
Other	2.0	4.7	5.3	3.7	3.9
**Total**	**100.0**	**100.0**	**100.0**	**100.0**	**100.0**

Supplement Table S8. Questionnaire n.1

Characteristics of the interviewee

Do you purchase FRESH FRUIT? ☐ yes ☐ no

If so: ☐ only for yourself ☐ only for others ☐ both

How often do you eat FRESH FRUIT (one answer only)?

Multiple times a day
Regularly, as a snack
Regularly, usually with meals
Occasionally
Never

Do you buy KIWI? ☐ yes ☐ no

If so: ☐ only for yourself ☐ only for others ☐ both

If yes, where do you prefer to purchase KIWIS (one answer only)?

directly from the producer
at specialized stores
at the supermarket/discount store
at the local market
other

How often do you eat KIWI (one answer only)?

More than once a day
Regularly, as a snack
Regularly, usually with meals
Occasionally
Never

As a rule, in which season do you eat KIWI (one answer only)?

The whole year
Preferably in summer
Preferably in winter
Other

Are you familiar with organic kiwis? ☐ yes ☐ no
If yes, do you purchase them? ☐ sì ☐ no
Are you familiar with yellow kiwis? ☐ yes ☐ no
If yes, do you purchase them? ☐ yes ☐ no

Are you familiar with red kiwis?	☐ yes ☐ no
If yes, do you purchase them?	☐ yes ☐ no

Supplement Table S9. Questionnaire n.2

**Attitude towards consuming fresh fruit and kiwi.**

With reference to the statements, you will find below, indicate your level of agreement/disagreement by expressing it on a scale from 1 to 4, where:

1 = Totally disagree
2 = Partially disagree
3 = Partially agree
4 = Totally agree

		**1 to 4**
1.	I like to eat fresh fruit.	
2.	For me, it's essential to eat fresh fruit every day.	
3.	I eat fresh fruit because it's good for my health.	
4.	Fresh fruit is a very healthy product.	
5.	Fresh fruit provides a multitude of nutrients.	
6.	I like to eat fresh fruit as it is, without any particular transformations.	
7.	I enjoy eating fresh fruit as an ingredient in cakes, fruit salads, smoothies, etc.	
8.	I'm willing to follow a diet entirely based on fruit for 1 day.	
9.	I'm willing to follow a diet entirely based on fruit for 1 week.	
10.	I usually buy fresh fruit only if I find it at a low price.	
11.	I usually don't buy fresh fruit because it's too expensive.	
12.	I like to eat kiwi.	
13.	I eat kiwi for its remarkable nutritional properties.	
14.	I eat kiwi for its high vitamin C content.	
15.	I eat kiwi (after dinner) because it improves the quality of my sleep.	
16.	I eat kiwi for its laxative effect.	
17.	I like to eat kiwi as it is.	
18.	I like to eat kiwi as an ingredient in cakes, fruit salads, smoothies, etc.	
19.	If green, yellow, and red kiwis are available, I choose the green ones.	
20.	If green, yellow, and red kiwis are available, I choose the yellow ones.	
21.	If green, yellow, and red kiwis are available, I choose the red ones.	
22.	I'm willing to pay 25% more than the usual price to taste a red kiwi.	
23.	I'm willing to pay 50% more than the usual price to taste a red kiwi.	
24.	I like to experiment with new foods and flavours, even if I don't know them.	
25.	If I don't know a food, I don't eat it.	

Supplement Text:

1. USING THE ATTITUDE TOWARDS KIWI IN CONJOINT EXPERIMENTS ANALYSIS

During the research we asked the people of the sample to say if they would buy (yes or not) a red kiwi characterized by different *attributes*: price (LOW, HIGH), provider (LOCAL, INTERNATIONAL), production (BIO, INTEGRATED) and quality certification (PRESENT, NOT PRESENT). We showed to the interviewed 6 different groups composed by 3 different kinds of red kiwi characterized by different *levels* of the *attributes*, and for each of them the interviewed must choose only one or none. This kind of data can be analyzed by a logistic regression model where the explanatory variables are the dummy of the *levels* for each *attribute* indicated above, and in particular:

LOW PRICE (=1 if 4 euros, 0 if 8 or 10 euros)

LOCAL PROVIDER (=1 if local, 0 if international)

BIO PRODUCTION (=1 if bio, 0 if integrated)

QUALITY CERTIFICATION PRESENT (=1, if present, 0 if absent)

We estimated a logistic regression model estimated for each of the following segments of the sample, formed using the attitude towards kiwi, previously estimated by Rasch model, as follows:

Supplement Table S10. Segment for the conjoint analysis and their characteristics

SEGMENT	Sample %	P: Probability of choosing at least 1 red kiwi in the 6 groups
1. Kiwi attitude <= -1	12.4	P <= 0.82
2. Kiwi attitude in the interval (-1,+1)	42.8	0.82 < P < 0.93
3. Kiwi attitude >= +1	44.8	P >= 0.93

The following graph (Figure 3) shows the probability of choosing at least 1 of the red kiwis proposed in the 6 groups, as function of the kiwi attitude, estimated by a logistic regression model, together with the distribution of attitude in the sample.

Supplement Figure S3. Probability of choosing at least 1 red kiwi in the 6 groups experiment and distribution of the kiwi attitude in the sample

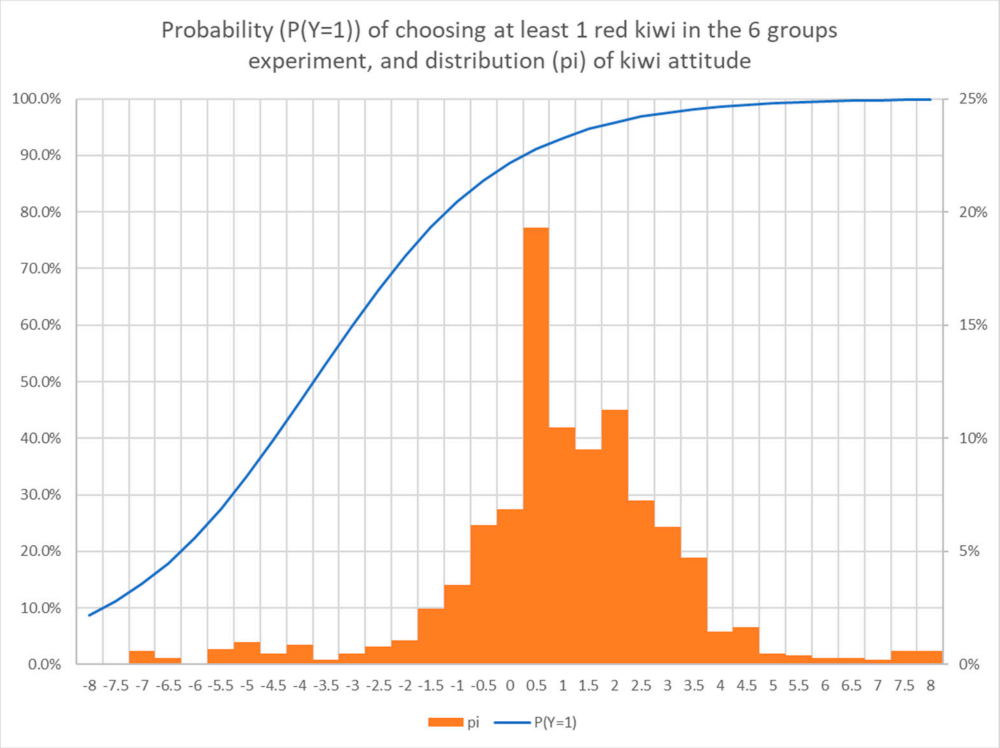

We then use the coefficients of logistic regression to perform a conjoint analysis, calculating the *Importance* score for each *attribute*, and the preference score for each *level,* for each segment. The following tables contains the coefficients of the logistic regression models, their p-value, the *Importance* score for each *attribute*, and preference score for each *level,* obtained applying a logistic regression model to the choice data for each of the 3 segments of Table 10.

Supplement Table S11 – Conjoint analysis for segment 1

Importance scores (attribute partworths) for red kiwi: segment Kiwi attitude <= -1

	Raw regression coefficient	p-value	Range	Importance score
LOW PRICE	3.018	0.000	3.018	85%
LOCAL PROVIDER	0.089	0.682	0.089	3%
BIO PRODUCTION	0.375	0.023	0.375	11%
QUALITY CERTIFICATION PRESENT	0.055	0.716	0.055	2%
Model intercept	-3.500	0.000		
**Total**			**3.537**	**100%**

Preference scores (level partworths) for red kiwi: segment Kiwi attitude <= -1

		Raw regression coefficient	Centered coefficient	Preference score
PRICE				
	8-10 euros	0.000	-1.51	-43%
	4 euros	3.018	1.51	43%
PROVIDER				
	International	0.000	-0.04	-1%
	Local	0.089	0.04	1%
PRODUCTION				
	Integreated	0.000	-0.19	-5%
	Bio	0.375	0.19	5%
QUALITY CERTIFICATION				
	Absent	0.000	-0.03	-1%
	Present	0.055	0.03	1%

Supplement Table S12 – Conjoint analysis for segment 2

Importance scores (attribute partworths) for red kiwi: segment Kiwi attitude in the interval (-1,+1)

	Raw regression coefficient	p-value	Range	Importance score
LOW PRICE	2.233	0.000	2.233	72%
LOCAL PROVIDER	0.477	0.000	0.477	15%
BIO PRODUCTION	0.352	0.000	0.352	11%
QUALITY CERTIFICATION PRESENT	-0.047	0.451	0.047	2%
Model intercept	-2.215	0.000		
**Total**			**3.108**	**100%**

Preference scores (level partworths) for red kiwi: segment Kiwi attitude in the interval (-1,+1)

		Raw regression coefficient	Centered coefficient	Preference score
PRICE				
	8-10 euros	0.000	-1.12	-36%
	4 euros	2.233	1.12	36%
PROVIDER				
	International	0.000	-0.24	-8%
	Local	0.477	0.24	8%
PRODUCTION				
	Integreated	0.000	-0.18	-6%
	Bio	0.352	0.18	6%
QUALITY CERTIFICATION				
	Absent	0.000	0.02	1%
	Present	-0.047	-0.02	-1%

Supplement Table S13 – Conjoint analysis for segment 3

Importance scores (attribute partworths) for red kiwi: segment Kiwi attitude >= +1

	Raw regression coefficient	p-value	Range	Importance score
**LOW PRICE**	1.932	0.000	1.932	58%
**LOCAL PROVIDER**	0.819	0.000	0.819	25%
**BIO PRODUCTION**	0.527	0.000	0.527	16%
**QUALITY CERTIFICATION PRESENT**	-0.035	0.535	0.035	1%
**Model intercept**	-2.108	0.000		
**Total**			**3.313**	**100%**

Preference scores (level partworths) for red kiwi: segment Kiwi attitude >= +1

		Raw regression coefficient	Centered coefficient	Preference score
**PRICE**				
	8-10 euros	0.000	-0.97	-29%
	4 euros	1.932	0.97	29%
**PROVIDER**				
	International	0.000	-0.41	-12%
	Local	0.819	0.41	12%
**PRODUCTION**				
	Integreated	0.000	-0.26	-8%
	Bio	0.527	0.26	8%
**QUALITY CERTIFICATION**				
	Absent	0.000	0.02	1%
	Present	-0.035	-0.02	-1%

As we may see in all segments the QUALITY CERTIFICATION is an attribute not significant in explaining the choices of the interviewed. This is a very important results because quality certification constitutes a big effort for producers which apparently, from this data, doesn't influence consumer choices.

The preference scores show that the consumers prefer to buy red kiwi if with LOW PRICE, LOCAL PROVIDER and BIO PRODUCTION.

The PRICE is the attribute with the highest importance, but this shows a decreasing value passing from the first segment (with lower attitude towards kiwi) (85%), to the third segment (with highest attitude towards kiwi) (58%).

The other attributes show an importance much lower, but while PROVIDER is not significant for the first segment, is significant and second attributes as importance in the other segments.

The PRODUCTION is the second factor of importance for the first segment, while is the third for the other.

In synthesis we may say that for lower level of kiwi attitude (that are only 12.4% of the sample) the price is the determinant attribute in the choice to buy a red kiwi, while for higher attitude levels (segment 2 and 3), that are most of the sample, the PROVIDER and PRODUCTION attributes play a certain role, much higher for segment 3 than for segment 2.

In segment 3, from Table 11, are comprised generally people of country 1 and 4 (Italy and France) of both sexes, all ages but with income greater or equal to the 4th class (>= 2500 euros). In segment 2 we may find all other kind of people. Therefore, if the producers want to sell red kiwi at high price, they must offer local and bio production to the high-income class in Italy and France. For other kind of consumers, otherwise, they must reduce the price, although local and bio production can play a relevant roll.
